# The efficacy of suprascapular nerve radiofrequency and intra-articular steroıd injectıon in frozen shoulder treatment: randomızed controlled trial

**DOI:** 10.1186/s12871-026-04162-7

**Published:** 2026-08-13

**Authors:** Dostali Ali̇yev, İbrahim Aşik

**Affiliations:** 1 Department of Anesthesiology and Reanimation, Section of Algology, Tobb Etü Hospital, Beştepe, Yaşam Cd. No:5 Yenimahalle, Ankara, 06560 Turkey; 2 Department of Anesthesiology and Reanimation, Faculty of Medicine, Section of Algology, Ankara University, Ankara, Turkey

**Keywords:** Frozen shoulder, Pulsed radio frequency, Intra-articular injection, Ultrasound-guided intervention, Steroids

## Abstract

**Background:**

This study aims to address the impacts of intra-articular steroid injection (IASI) alone versus the combination of suprascapular nerve (SSN) pulsed radiofrequency (PRF) and IASI on pain relief and improvement in shoulder range of motion (ROM) in patients with adhesive capsulitis (AC) (frozen shoulder).

**Methods:**

This study included 80 patients with AC. Participants were randomly assigned to two groups. A combination of suprascapular nerve pulsed radiofrequency and IASI were administered to the first group and IASI to the second.

Both groups underwent a standardized physical therapy program for three weeks following the intervention. Outcome measures included active and passive ROM (AROM and PROM), Visual Analog Scale (VAS) for pain, and the Shoulder Pain and Disability Index (SPADI), assessed at baseline and 4th week, and 12th week post-treatment.

**Results:**

80 patients (40 patients in each group) completed this study. Both interventions were found to be effective in both groups (*p* < 0.05). However, intergroup comparisons revealed that patients in the SNNR group experienced significantly better improvements in pain levels (as measured by VAS and SPADI scores) and ROM (*p* < 0.05). No post-procedural complications were observed in either group.

**Conclusions:**

This study revealed that the administration of PRF lesioning of the SSN utilizing a UG method combined with intra-articular steroid injection yielded better and faster pain relief, reduced disability, and better AROM and PROM in comparison to intra-articular injection alone in AC patients.

**Trial registration:**

This study was retrospective registered with the U.S. Clinical Trial Registry (U.S. Clinical Trial Registry No NCT06778577, date 20241125).

**Supplementary Information:**

The online version contains supplementary material available at https://doi.org/10.1186/s12871-026-04162-7.

## What is known

- Adhesive capsulitis is commonly treated with physical therapy and intra-articular steroid injections, which provide short- to mid-term pain relief and functional improvement.
- Suprascapular nerve interventions, including blocks and pulsed radiofrequency, have been shown to be effective for shoulder pain management, but are less frequently incorporated into standard treatment algorithms.

## What is new

- This randomized controlled trial demonstrates that ultrasound-guided suprascapular nerve pulsed radiofrequency combined with intra-articular steroid injection provides superior pain relief and greater improvement in shoulder range of motion compared with intra-articular injection alone.
- The findings highlight the added clinical value of neuromodulatory treatment targeting the suprascapular nerve in frozen shoulder management without increasing complications.

## Introduction

Adhesive capsulitis (frozen shoulder) is a condition characterized by severe pain and restricted motion in the shoulder (glenohumeral joint). These symptoms can substantially impair daily functioning and reduce quality of life [1].

The primary goals of treatment include pain relief, improved joint mobility, and enhancement of the patient’s overall quality of life. Commonly employed treatment modalities include physical therapy, analgesics, intra-articular injections, and suprascapular nerve blocks. Surgical intervention is typically reserved for cases that are refractory to conservative management [2, 3].

In adhesive capsulitis, pain arises from both inflammatory changes within the glenohumeral joint capsule and altered neural pain processing. Intra-articular steroid injection is an established treatment that reduces synovial inflammation and provides short- to medium-term pain relief, thereby facilitating participation in rehabilitation programs [2, 3]. In contrast, suprascapular nerve pulsed radiofrequency produces prolonged neuromodulatory analgesia without causing neural destruction and has shown promising results in patients with chronic shoulder pain and adhesive capsulitis [4]. Given their distinct but complementary mechanisms of action, combining SSN-PRF with IASI may provide greater pain relief and functional improvement than either intervention alone by targeting both inflammatory and neural components of pain.

Intra-articular steroid injection is widely used to reduce inflammation and pain in adhesive capsulitis, whereas suprascapular nerve pulsed radiofrequency may provide prolonged neuromodulatory analgesia [5].Combining these two modalities may offer synergistic benefits by targeting both inflammatory and neural pain mechanisms.

This study aims to address the impacts of intra-articular steroid injection (IASI) alone with those of a combined treatment involving suprascapular nerve pulsed radiofrequency (SSN-PRF) and IASI on pain relief and range of motion (ROM) in adhesive capsulitis (AC) (frozen shoulder) patients.

## Materials and methods

This study was carried out in a single center in Ankara - Türkiye between 15.08.2021 and 20.10.2022 upon the approval from the ethics committee (Ankara University, Faculty of Medicine, Human Research Ethics Committee, IRB number: İ7-492-21, date: 12.08.2021). This study was retrospective registered with the U.S. Clinical Trial Registry (U.S. Clinical Trial Registry No: NCT06778577, date: 2024-11-25). The Declaration of Helsinki was followed throughout the study process, and confidentiality was guaranteed for the patient data. All participants provided written informed consent after receiving detailed information about the study.

The primary outcome of this study was the change from baseline in Visual Analog Scale (VAS) pain score at the 12th week after intervention, reflecting sustained pain relief. Secondary outcomes included VAS pain scores at the 4th week, Shoulder Pain and Disability Index (SPADI) scores, and active and passive ROM measurements at follow-up time points (Table 1).

**Table 1:** Schedule of enrolment, interventions, and outcome assessments

Study procedures	Screening	Baseline (Week 0)	Weeks 1-3	Week 4	Week 12
Eligibility assessment	✓				
Written informed consent	✓				
Demographic and clinical characteristics		✓			
Medical history and physical examination		✓			
MRI evaluation*		✓			
Randomization		✓			
Ultrasound-guided intervention (SSN-PRF + IASI or IASI)		✓			
Standardized physiotherapy†			✓		
Visual Analog Scale (VAS)		✓		✓	✓
Shoulder Pain and Disability Index (SPADI)		✓		✓	✓
Active range of motion (AROM)		✓		✓	✓
Passive range of motion (PROM)		✓		✓	✓
Adverse event assessment‡		✓		✓	✓
Review of concomitant treatments				✓	✓

*Abbreviations*: *SSN-PRF* Suprascapular nerve pulsed radiofrequency, *IASI* Intra-articular steroid injection, *VAS* Visual analog scale, *SPADI* Shoulder pain and disability index, *AROM* Active range of motion, *PROM* Passive range of motion

†Following the intervention, all participants completed a standardized physiotherapy program consisting of 15 supervised sessions (5 sessions per week for 3 consecutive weeks)

‡Adverse events, including infection, hematoma, motor weakness, sensory disturbance, and prolonged pain exacerbation, were evaluated immediately after the procedure and at each scheduled follow-up visit

**Fig. 1:**
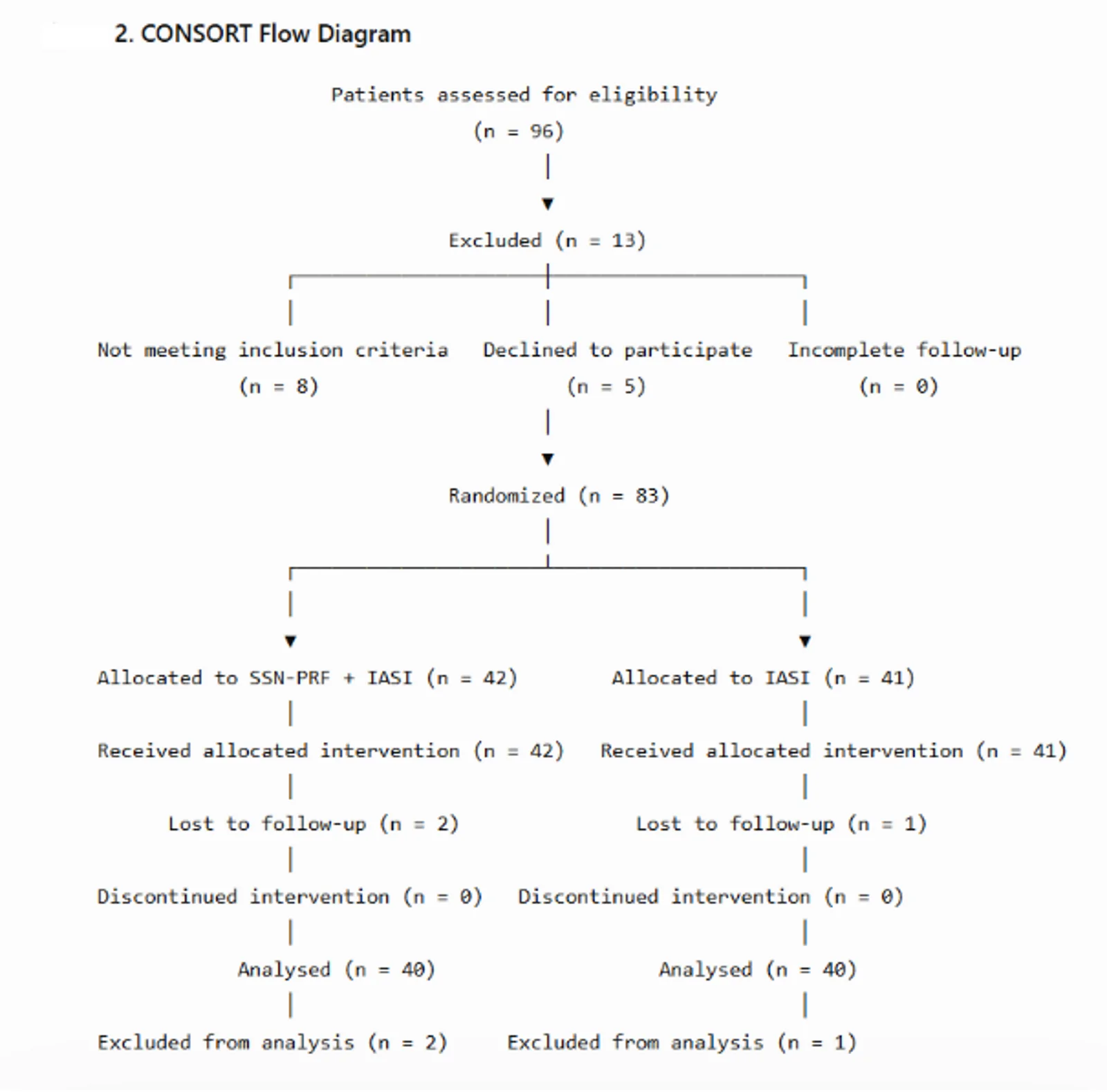
CONSORT flow diagram

A total of 96 patients diagnosed with frozen shoulder were assessed for eligibility. Of these, 8 patients did not meet the inclusion criteria, 5 declined to participate, and 3 were excluded due to incomplete follow-up data. Consequently, a total of 80 patients were included in the study and randomized (Fig. 1). Patients were recruited consecutively from the Pain Medicine (Algology) outpatient clinic of Ankara University Hospital. Adhesive capsulitis was initially diagnosed clinically based on pain, restricted active and passive ROM, and physical examination findings [6].

Diagnosis was established by an experienced pain physician with more than 10 years of musculoskeletal practice. MRI was used primarily to support the diagnosis and exclude alternative shoulder pathologies such as rotator cuff tears, labral lesions, osteoarthritis, and neoplastic lesions. MRI findings considered supportive of adhesive capsulitis included capsular thickening, particularly within the axillary recess, and thickening of the coracohumeral ligament [7].

The radiologist evaluating MRI findings was blinded to treatment allocation.

Inclusion criteria include age of > 20 years, unilateral shoulder involvement, ROM restriction ≥ 50% persisting for over 3 months, and baseline VAS ≥ 4. ROM restriction was assessed in flexion, abduction, external rotation, and internal rotation, and a reduction of at least 50% in one or more planes compared with expected normal values was required. Most participants presented with pain-predominant adhesive capsulitis, reflected by baseline VAS scores ≥ 4 and persistent symptoms for more than 3 months.

Exclusion criteria included a history of degenerative shoulder diseases (e.g., rheumatoid arthritis, osteoarthritis, labral or articular cartilage lesions, malignancy), prior shoulder trauma and surgery, neurologic disorders (neurological disorders affecting shoulder function, including stroke, traumatic brain injury, cervical radiculopathy and peripheral neuropathy), cervical spine pathology, known drug allergies, pregnancy or lactation, prior intra-articular injections (steroids or hyaluronic acid) within the past 4 weeks, and baseline VAS score < 4. Patients who had received suprascapular nerve block within the previous 3 months were excluded.

Patients were randomly assigned to study groups using a computer-generated randomization sequence. The first group received a PRF lesioning of the SSN+IASI and the second group received IASI. All interventions were conducted under sterile conditions and using ultrasound.

Randomization was performed using a computer-generated random number sequence with a 1:1 allocation ratio. Allocation concealment was ensured using sealed envelopes prepared by an independent staff member. Outcome assessors were blinded to group allocation.

All patients in both groups participated in a physical therapy program for a duration of three weeks following the intervention. All patients were instructed to avoid another shoulder pain treatments, including analgesics, acupuncture, or injections throughout the study duration. All procedures in both groups were performed by the same physician under ultrasound guidance.

Due to the interventional nature of the procedures, participant and provider blinding was not feasible. However, all clinical assessments, including baseline and follow-up evaluations, were performed by the same blinded assessor to minimize assessment bias. The assessor had more than 10 years of experience in musculoskeletal and pain medicine assessments.

**Fig. 2:**
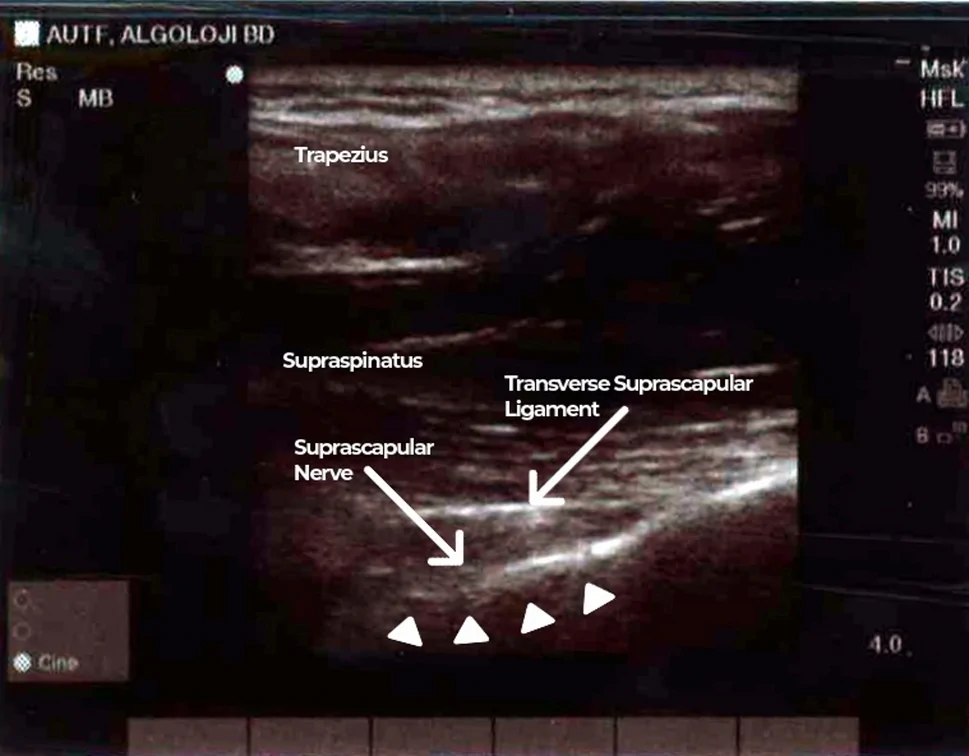
Ultrasonographic image of the suprascapular nerve and surrounding tissues

SNNR group: The location of the SSN was determined using a linear ultrasound probe via a posterior approach, beneath the transverse suprascapular ligament, following sensory stimulation (50–100 Hz, 1-ms pulse width, ≤ 0.5 volts) and motor stimulation (2 Hz, 1-ms pulse width, up to 1 V) (Fig. 2).

Once the nerve was localized, pulsed radiofrequency (PRF) applied at 42 °C for 180 s with a pulse frequency of 2 Hz, consistent with neuromodulatory rather than neurodestructive intent. Following the radiofrequency procedure, an intra-articular steroid injection was administered, consisting of a combination of 40 mg triamcinolone and 3 mL of 0.5% bupivacaine hydrochloride. The intra-articular injection was performed using the same ultrasound-guided posterior approach described for the IACI group.

IACI Group: The patients in the IACI group received intraarticular 40 mg of triamcinolone and 3 mL of 0.5% bupivacaine shoulder joint injection technique used by Chen et al.was applied [4]. The patients received the injections while sitting on a chair. Specifically, the probe used for injection was positioned along the musculotendinous junction of the infraspinatus muscle to visualize the posterior labrum and humeral head, and the lateral approach was used. The injection site was at the joint capsule, slightly lateral to the posterior labrum (Fig. 2.).

Both groups participated in a standardized physical therapy program for three weeks following the intervention. The physical therapy program consisted of supervised stretching and range-of-motion exercises performed five days per week for three weeks (fifteen supervised physiotherapy sessions (5 sessions/week for 3 weeks). Compliance was monitored through physiotherapy records.

All clinical assessments were performed by the same physician with more than 10 years of experience in musculoskeletal and pain medicine assessments. The primary outcome measure was the reduction in pain intensity, assessed using the VAS.

Secondary clinical outcome measures include the SPADI score, a validated, self-reported questionnaire utilized when measuring shoulder disorder-related pain (5 items) and disability (8 items). The pain subscale ranges between 0 and 50, whereas the disability subscale ranges between 0 and 80, with the total scale between 0 and 130. Higher scores indicate a higher level of pain or disability [8].

The secondary outcome, ROM measurements were obtained using a universal goniometer following a standardized protocol. Flexion and abduction were measured with patients in the standing position. Internal and external rotation were measured with the shoulder in 90° abduction and the elbow flexed to 90°. Internal and external rotation were measured with the patient in the supine position, with the shoulder abducted to 90° and the elbow flexed to 90°. Internal and external rotation were measured at the maximum pain-free abduction achievable by the patient. When full 90° abduction was not possible, measurements were performed at the highest tolerated abduction angle while maintaining a standardized position. The forearm was moved posteriorly for external rotation and anteriorly for internal rotation, and the range of motion was measured using a universal goniometer. Measurements were performed using a universal goniometer according to standardized musculoskeletal assessment guidelines [9]. Each measurement was performed twice, and the mean value was recorded. All assessments were conducted by the same experienced physician to ensure consistency.

Adverse events including infection, bleeding, motor weakness, sensory disturbances, and prolonged pain exacerbation were actively assessed during follow-up visits (Table 1).

Statistical Analyses.

Statistical analyses were performed utilizing SPSS version 12.0 for Windows. The Kolmogorov-Smirnov test was applied to assess the distribution of data in both groups. Continuous demographic variables were evaluated with the Mann-Whitney U test, while categorical variables were analyzed using the chi-square (χ²) test. For within-group comparisons, the Wilcoxon signed-rank test was employed. Measurements taken during follow-up were compared to baseline values, and differences between groups were also analyzed utilizing the Mann-Whitney U test. Statistical significance was set at *p* < 0.05. Because the outcome variables were not normally distributed according to the Kolmogorov–Smirnov test and the sample size was relatively modest, nonparametric methods were selected for the primary analyses. Within-group changes over time were assessed using the Wilcoxon signed-rank test, whereas between-group comparisons at each predefined follow-up time point were performed using the Mann–Whitney U test. Although repeated-measures ANOVA or linear mixed-effects models may provide a more comprehensive analysis of longitudinal data, the predefined objective of this study was to compare treatment groups at clinically relevant follow-up visits using robust nonparametric methods.

### Data availability

The data that support the findings of this study are available on request from the corresponding author.

## Results

Table 2 presents the physical characteristics of groups. There was no significant difference in any of the baseline characteristics, VAS or SPADI scores, AROM, or PROM.

**Table 2:** Demographic and clinical characteristics of patients

Descriptors		SSNR Group (*n* = 40)				IACI Group (*n* = 40)			
		*n*		%		*n*	%		*P*
Gender	Female	37		48,7		15	39,5		
	Male	39		51,3		23	60,5		,742
Total		40		100		40	100		
Age	mean + SD	55.0 ± 9.2 (min 20 max 81)				57.1 ± 10.9 (min 20 max 78)			,588
Weight (kg)	mean + SD	75,97 + 14,72 (min 44 max 110)				78,74 + 13,97 (min 49 max 107)			,297
Height (cm)	mean + SD	169,70 + 8,21 (min 150 max 190)				169,92 + 9,81 (min 150 max 190)			,886
BMI (kg/m2)	mean + SD	26,53 + 5,30 (min 17,4 max 43,7)				27,36 + 4,30 (min 21 max 34,9)			,509
Pain duration (month)		5.1 ± 3.1				5 ± 3.4			,654
VAS		6.5 ± 1.3				6.4 ± 1.5			0.990
SPADI score									
Pain		62.1 ± 15.6				56.8 ± 14.5			0.283
Disability		51.3 ± 11.5				50.2 ± 20.5			0.497
Total		55.4 ± 11.8				52.16 ± 125			0.450
AROM									
Flexion		124.8 ± 19.9				128.5 ± 13.6			0.614
Extension		32.4 ± 10.3				36.4 ± 12.8			0.605
Abduction		82.2 ± 23.3				87.6 ± 20.9			0.358
External rotation		34.6 ± 13.2				38.9 ± 16.2			0.413
Internal rotation		43.3 ± 15.4				45.5 ± 5.6			0.696
PROM									
Flexion		126.8 ± 19.8				130.5 ± 13.2			0.620
Extension		34.4 ± 10.5				38.4 ± 12.8			0.611
Abduction		84.2 ± 21.3				88.5 ± 21.5			0.352
External rotation		36.6 ± 14.2				39.8 ± 16.2			0.418
İnternal rotation		45.3 ± 15.1				48.5 ± 5.5			0.689

Values are presented as mean ± SD, number (%);

*SSNR* Suprascapular nerve radiofrequency, *IACI *Intra articular inejction, *VAS* Visual analog scale, *SPADI* Shoulder pain and disability index, *BMI *Body mass index, *SD S*tandart deviation

Table 3 presents the patients’ pre- and post-procedural pain scores and joint ROM measurements.

**Table 3:** All the Measured Variables Before and After Treatment

	SSNR Group (*n* = 40)	IACI Group (*n* = 40)	
	Mean ± SDP value	Mean ± SDP value	*P* value
VAS baseline	6.5 ± 1.3	6.4 ± 1.5	0.990
VAS (4th wk)	2.1 ± 1.5 < 0.001	3.7 ± 2.1 < 0.001	**0.012**
VAS (12th wk)	1.4 ± 1.1 < 0.001	3.0 ± 2.1 < 0.001	**0.023**
Pain SPADI baseline	62.1 ± 15.6	56.8 ± 14.5	0.283
Pain SPADI (4th wk)	21.4 ± 12.4 < 0.001	42.3 ± 22.30.002	**0.001**
Pain SPADI (12th wk)	17.1 ± 14.1 < 0.001	37.1 ± 22.1 < 0.001	**0.002**
Disability SPADI baseline	51.3 ± 11.5	50.2 ± 20.5	0.497
Disability SPADI (4th wk)	19.4 ± 12.1 < 0.001	37.5 ± 18.60.008	**0.001**
Disability SPADI (12th wk)	14.0 ± 12.0 < 0.001	33.2 ± 17.0 < 0.001	**< 0.001**
Total SPADI baseline	55.4 ± 11.8	**52.16 ± 12.5**	0.450
Total SPADI (4th wk)	20.1 ± 11.3 < 0.001	39.3 ± 19.30.005	**0.002**
Total SPADI (12th wk)	14.6 ± 12.1 < 0.001	35.2 ± 18.5 < 0.001	**< 0.001**
Active FL baseline	124.8 ± 19.9	128.5 ± 13.6	0.614
Active FL (4th wk)	153.9 ± 16.9 < 0.001	145.4 ± 10.3 < 0.001	**0.020**
Active FL (12th wk)	160.2 ± 13.7 < 0.001	150.2 ± 10.5 < 0.001	**0.010**
Active EX baseline	32.4 ± 10.3	36.4 ± 12.8	0.605
Active EX (4th wk)	51.7 ± 10.1 < 0.001	44.7 ± 14.30.007	0.108
Active EX (12th wk)	54.1 ± 10.6 < 0.001	45.3 ± 12.30.006	**0.019**
Active Abd baseline	82.2 ± 23.3	87.6 ± 20.9	0.358
Active Abd (4th wk)	116.5 ± 25.7 < 0.001	110.8 ± 22.3 < 0.001	0.345
Active Abd (12th wk)	131.2 ± 27.1 < 0.001	117.6 ± 16.9 < 0.001	0.091
Active Intr. Rot baseline	43.3 ± 15.4	45.5 ± 5.6	0.696
Active Intr. Rot (4th wk)	69.3 ± 11.7 < 0.001	60.9 ± 8.2 < 0.001	**0.005**
Active Intr. Rot (12th wk)	75.2 ± 10.5 < 0.001	66.0 ± 6.3 < 0.001	**0.002**
Active Extr. Rot baseline	34.6 ± 13.2	38.9 ± 16.2	0.413
Active Extr. Rot (4th wk)	56.3 ± 14.6 < 0.001	52.7 ± 17.8 < 0.001	0.772
Active Extr. Rot (12th wk)	62.7 ± 12.4 <0.001	59.8 ± 16.0 <0.001	0.427
Passive FL baseline	126.8 ± 19.8	130.5 ± 13.2	0.620
Passive FL (4th wk)	158.9 ± 15.9 < 0.001	147.4 ± 10.3 < 0.001	**0.019**
Passive FL (12th wk)	165.2 ± 12.7 < 0.001	154.2 ± 10.5 < 0.001	**0.011**
Passive EX baseline	34.4 ± 10.5	38.4 ± 12.8	0.611
Passive EX (4th wk)	56.5 ± 10.1 < 0.001	45.7 ± 14.20.007	0.098
Passive EX (12th wk)	58.1 ± 10.6 < 0.001	47.3 ± 12.30.006	**0.015**
Passive Abd baseline	84.2 ± 21.3	88.5 ± 21.5	0.352
Passive Abd (4th wk)	117.5 ± 24.7 < 0.001	110.4 ± 22.3 < 0.001	0.341
Passive Abd (12th wk)	132.2 ± 26.1 < 0.001	119.6 ± 16.9 < 0.001	0.090
Passive Intr. Rot baseline	45.3 ± 15.1	48.5 ± 5.5	0.689
Passive Intr. Rot (4th wk)	70.3 ± 10.7 < 0.001	59.9 ± 8.2 < 0.001	**0.006**
Passive Intr. Rot (12th wk)	77.2 ± 10.1 < 0.001	67.3 ± 6.1 < 0.001	**0.003**
Passive Extr. Rot baseline	36.6 ± 14.2	39.8 ± 16.2	0.418
Passive Extr. Rot (4th wk)	57.3 ± 14.1 < 0.001	53.7 ± 17.4 < 0.001	0.769
Passive Extr. Rot (12th wk)	63.8 ± 12.1 <0.001	60.8 ± 15.8 <0.001	0.424

Values are presented as mean ± SD, number (n)

*SSNR* Suprascapular nerve radiofrequency, *IACI *Intra articular inejction, *VAS* Visual analog scale, *SPADI *Shoulder pain and disability index, *WK* Week, *FL *Flexion, *EX *Extension, *Abd *Abduction, *Intr. Rot *Internal rotation, *Extr. Rot *External rotation, *SD S*tandart deviation

In both groups, the reductions in pain and the increases in ROM were statistically significant (*p* < 0.001).

Comparing the groups, significant improvements were found in the SNNR group in the VAS and SPADI scores, in AROM (active flexion:4th and 12th weeks; active extension in 12th week, and internal rotation in 4th and 12th weeks; all *p* < 0.05 for all) and PROM (passive flexion: 4th and 12th weeks, passive extension in 12th week, and internal rotation in 4th and 12th weeks; *p* < 0.05 for all). No cases of infection, hematoma, motor weakness, sensory deficit, or other procedure-related adverse events were observed during follow-up. None of the patients received any additional treatment (other than regular physical therapy) throughout the study process.

## Discussion

In this study, the efficacy of the combination treatment of suprascapular nerve radiofrequency (RF) and IASI was compared with IASI alone in AC patients. Results indicate that the combined treatment was more effective in both pain reduction and improvement of ROM.

AC is one of the most challenging painful conditions encountered in pain clinics. The two most prominent clinical features are reduced joint ROM and severe pain. Frozen shoulder may present as a primary (idiopathic) condition or as a manifestation of an underlying systemic disease [10, 11]. Although adhesive capsulitis most commonly affects individuals between 40 and 60 years of age, younger adults may also develop the condition, particularly in the presence of prolonged shoulder immobilization, trauma, surgery, or idiopathic causes. In the present study, although the minimum participant age was 20 years, the overall age distribution was consistent with the typical epidemiology of adhesive capsulitis (mean age: 55.0 ± 9.2 years in the SSN-PRF group and 57.1 ± 10.9 years in the IASI group) [5]. Therefore, the inclusion of a small number of younger patients is unlikely to have influenced the overall findings or their generalizability.

Physical therapy and intra-articular corticosteroid injections (IACI) are among the most frequently used treatment modalities for frozen shoulder. These treatments are often applied in combination [2, 3]. IACI contributes to pain control and reduces inflammation within the joint, thereby enhancing the effectiveness of physical therapy. Physical therapy improves muscle strength and helps restore the ROM of the shoulder joint [12].

In a study carried out by Kayaokay et al., patients with shoulder joint disorders who received local steroid injections experienced earlier recovery compared to those in the physical therapy group [13]. Another study also found steroid injections to be effective in the treatment of shoulder disorders [14].

Intra-articular triamcinolone was used in this study. Triamcinolone is thought to exert its anti-inflammatory and analgesic effects through multiple mechanisms. These include reduced synovial blood flow, alterations in synovial fluid composition, suppression of leukocyte gene expression, and changes in collagen synthesis. Branched esterification and the microcrystalline formulation contribute to a more potent and prolonged effect at the injection site [15, 16].

In recent years, studies demonstrated the effectiveness of suprascapular nerve pulsed radiofrequency (PRF) in relieving pain associated with AC. The efficacy of PRF in chronic pain was first reported by Sluijter et al. and its application to the suprascapular nerve was first performed in 2002 [17, 18]. Due to its non-destructive nature, safety, repeatability, and ability to provide long-term pain relief across different etiologies, PRF has become a widely used treatment option [19–21].

The exact action mechanism of pulsed radiofrequency remains unclear. It is hypothesized to exert its effects through modification of cell membrane properties, inhibition of C and A-δ fibers, and increased c-Fos immunoreactivity in the dorsal horn of the spinal cord [22–25].

In a randomized controlled trial involving 40 patients diagnosed with frozen shoulder, Abo Elfadl et al. found that the group receiving RF treatment achieved faster and more effective outcomes compared to the medical treatment group [26]. Another randomized study with 60 patients demonstrated that the suprascapular nerve PRF group had superior pain control and greater improvement in ROM compared to the group that received only a nerve block [27].

The magnitude of improvement observed in our study further supports these findings. At 12 weeks, the SSN-PRF + IASI group demonstrated a mean reduction of 5.1 points in VAS pain score (6.5 to 1.4), whereas the IASI group improved by 3.4 points (6.4 to 3.0). Similarly, the total SPADI score improved by 40.8 points in the combined treatment group (55.4 to 14.6) compared with 17.0 points in the IASI group (52.2 to 35.2). These findings suggest that adding SSN-PRF to intra-articular steroid injection provides greater reductions in pain and disability than corticosteroid injection alone.

The superior clinical outcomes observed with the combined treatment are biologically plausible. Intra-articular corticosteroid injection primarily suppresses synovial inflammation within the glenohumeral capsule, whereas pulsed radiofrequency modulates nociceptive transmission through the suprascapular nerve without producing neural destruction. Simultaneous targeting of inflammatory and neural pain pathways may provide more sustained analgesia, thereby facilitating greater participation in physiotherapy and leading to larger improvements in shoulder function and range of motion. This complementary mechanism has also been proposed in recent reviews of radiofrequency treatment for chronic shoulder pain [28].

Suprascapular nerve block and PRF procedures are often performed using CT or fluoroscopy guidance. However, ultrasound is more effective for visualizing the suprascapular nerve [29]. In this study, ultrasound guidance was used, and no complications were observed during or after the procedure.

Beyond statistical significance, the observed improvements also appear to be clinically meaningful. Previous studies have reported a minimal clinically important difference (MCID) of approximately 1.4–2.0 points for pain measured with the Visual Analog Scale and approximately 13 points for the Shoulder Pain and Disability Index (SPADI) in patients with shoulder disorders. Both treatment groups exceeded these thresholds; however, the combined SSN-PRF + IASI group achieved substantially greater improvements, supporting the clinical relevance of the additional benefit provided by suprascapular nerve pulsed radiofrequency.

The limitations of this study include a small sample size and short follow-up period. The lack of participant and provider blinding may have introduced performance or expectation bias, particularly for subjective outcome measures such as VAS and SPADI. Another limitation of this study is that baseline data on several established risk factors for adhesive capsulitis, including diabetes mellitus, thyroid disorders, previous history of adhesive capsulitis, and hand dominance, were not systematically collected. Consequently, the potential influence of these factors on treatment response could not be evaluated. Future studies should incorporate these variables to allow more comprehensive subgroup analyses and improve the interpretation and generalizability of the findings. Another limitation of this study is that repeated longitudinal outcomes were analyzed using nonparametric pairwise methods rather than mixed-effects modeling or repeated-measures approaches, which may better account for within-subject correlations over time and permit formal assessment of group × time interactions. In addition, no a priori power analysis was performed, which should be addressed in future confirmatory trials.

## Conclusions

Although various studies in the literature compared different treatment modalities for frozen shoulder, such as suprascapular nerve block, IASIs, and local anesthetic injections, to the best of our knowledge, this study revealed that the combination of SNN PRF with IASI was effective in both pain relief and improving joint ROM.

## Supplementary Information

Supplementary Material 1.

Supplementary Material 2.

Supplementary Material 3.

Supplementary Material 4.

Supplementary Material 5.

## Data Availability

The data that support the findings of this study are available on request from the corresponding author.

## Abbreviations

IASI: Intra-articular steroid injection
SSN: Suprascapular nerve
PRF: Pulsed radiofrequency
AC: Adhesive capsulitis (frozen shoulder)
AROM: Active range of motion
PROM: Passive range of motion
VAS: Visual Analog Scale
SPADI: The Shoulder Pain and Disability Index
