# Supplementary material for: The efficacy of suprascapular nerve radiofrequency and intra-articular steroıd injectıon in frozen shoulder treatment: randomızed controlled trial
Source: BMC Anesthesiol. 2026 Aug 13;26:663. doi: 10.1186/s12871-026-04162-7 (PMC13749883; doi:10.1186/s12871-026-04162-7)
Supplement: Supplementary file 1 [file 12871_2026_4162_MOESM2_ESM.pdf]

CERTIFICATE OF TRANSLATION AND PROOFREADING

THE MANUSCRIPT TITLED

THE EFFICACY OF SUPRASCAPULAR NERVE RADIOFREQUENCY AND INTRA-ARTICULAR STEROID INJECTION IN FROZEN SHOULDER TREATMENT

has been translated by our professional translators and proofread by two different native-speaker editors.
This certificate has been issued upon the request of the author.

7/5/2025

ÇEVİRİONLİNE TERCÜME
VOLKAN SERİN
Turgut Reis Mh. Mithatpaşa Cd. No:279/1
Tel: 0 850 840 32 45 Konak - İZMİR
Konak V.D. 172 520 57256

Volkan SERİN,
General Manager

ÇeviriOnline Translation
Adalet Mahallesi, Manas Bulvarı, Folkart Towers, B Kule, K:25, Kapı No:2511 Bayraklı - İzmir
ceviri@cevirionline.com
