# Supplementary material for: The efficacy of suprascapular nerve radiofrequency and intra-articular steroıd injectıon in frozen shoulder treatment: randomızed controlled trial
Source: BMC Anesthesiol. 2026 Aug 13;26:663. doi: 10.1186/s12871-026-04162-7 (PMC13749883; doi:10.1186/s12871-026-04162-7)
Supplement: Supplementary file 2 [file 12871_2026_4162_MOESM4_ESM.pdf]

İNSAN ARAŞTIRMALARI ETİK KURULU KARAR FORMU

ETİK KURULUN ADI	ANKARA ÜNİVERSİTESİ TIP FAKÜLTESİ İNSAN ARAŞTIRMALARI ETİK KURULU
AÇIK ADRES	Ankara Üniversitesi Tıp Fakültesi Morfoloji Binası 06100 Sıhhıye/ANKARA
TELEFON	0312 595 82 27
FAKS	0312 310 63 70
E-POSTA	tipinsanetik@ankara.edu.tr

BAŞVURU BİLGİLERİ	ARAŞTIRMANIN AÇIK ADI	Donuk Omuzda Ultrason Eşliğinde Uygulanan Supraskapular Sinir Pulsed Radyofrekans Nöromodulasyon ve Eklem içi Steroid Enjeksiyon Tedavi Etkinliğinin Araştırılması	
	KOORDİNATÖR/SORUMLU ARAŞTIRMACI ÜNVANI/ADI/SOYADI	Prof. Dr. İbrahim AŞIK	
	PROJE YÜRÜTÜCÜSÜ UNVANI/ADI/SOYADI (TÜBİTAK vb. gibi kaynaklardan destek alanlar için)		
	KOORDİNATÖR/SORUMLU ARAŞTIRMACININ UZMANLIK ALANI	Algoloji	
	KOORDİNATÖR/SORUMLU ARAŞTIRMACININ BULUNDUĞU MERKEZ	Ankara Üniversitesi Tıp Fakültesi Anesteziyoloji ve Reanimasyon Anabilim Dalı, Algoloji Bilim Dalı	
	ARAŞTIRMAYA KATILAN MERKEZLER	TEK MERKEZ ☒	ÇOK MERKEZLİ ☐

KARAR BİLGİLERİ	Karar No:İ7-492-21	Tarih: 12 Ağustos 2021
	Yukarıda bilgileri verilen başvuru dosyası ile ilgili belgeler araştırmanın/çalışmanın gerekçe, amaç, yaklaşım ve yöntemleri dikkate alınarak incelenmiş ve uygun bulunmuş olup araştırmanın/çalışmanın başvuru dosyasında belirtilen merkezde gerçekleştirilmesinde etik ve bilimsel sakınca bulunmadığına toplantıya katılan etik kurul üye tam sayısının salt çoğunluğu ile karar verilmiştir.	

İNSAN ARAŞTIRMALARI ETİK KURULU

ÇALIŞMA ESASI	İyi Klinik Uygulamaları Kılavuzu
BAŞKANIN UNVANI / ADI / SOYADI:	Prof.Dr.Nuray YAZIHAN

Unvanı/Adı/Soyadı	Uzmanlık Alanı	Kurumu	Araştırma ile ilişki		İmza
Prof.Dr.Nuray YAZIHAN	Fizyopatoloji	A.Ü.Tıp Fakültesi	E ☐	H ☒	
Prof.Dr.Serenay ELGÜN ÜLKAR	Tıbbi Biyokimya	A.Ü.Tıp Fakültesi	E ☐	H ☒	—
Prof.Dr.Hakan ERGÜN	Tıbbi Farmaloloji	A.Ü. Tıp Fakültesi	E ☐	H ☒	
Prof.Dr.Hatice ILGIN RUHİ	Tıbbi Genetik	A.Ü. Tıp Fakültesi	E ☐	H ☒	
Prof.Dr.Sevim AYDIN	Histoloji ve Embriyoloji	A.Ü. Tıp Fakültesi	E ☐	H ☒	
Prof.Dr.Berna SAVAŞ	Tıbbi Patoloji	A.Ü. Tıp Fakültesi	E ☐	H ☒	
Prof.Dr.Yasemin YAVUZ	Biyoistatistik	A.Ü. Tıp Fakültesi	E ☐	H ☒	
Prof.Dr.Deniz BALCI	Genel Cerrahi	A.Ü. Tıp Fakültesi	E ☐	H ☒	—
Prof.Dr.M.Kürşat GÖKCAN	Kulak, Burun ve Boğaz Hastalıkları	A.Ü. Tıp Fakültesi	E ☐	H ☒	
Doç.Dr.Yüksel ÜRÜN	Tıbbi Onkoloji	A.Ü.Tıp Fakültesi	E ☐	H ☒	
Doç.Dr.Cihangir AKYOL	Genel Cerrahi	A.Ü. Tıp Fakültesi	E ☐	H ☒	
Doç.Dr.Başak Ceyda MEÇO	Anesteziyoloji ve Reanimasyon	A.Ü. Tıp Fakültesi	E ☐	H ☒	
Doç.Dr.Halil ÖZDEMİR	Çocuk Sağlığı ve Hastalıkları	A.Ü. Tıp Fakültesi	E ☐	H ☒	
Doç.Dr.Emel OKULU	Çocuk Sağlığı ve Hastalıkları	A.Ü. Tıp Fakültesi	E ☐	H ☒	—
Doç.Dr.Mustafa Volkan KAVAS	Tıp Tarihi ve Etik	A.Ü. Tıp Fakültesi	E ☐	H ☒	—

Funda BAYKAL KILIÇ
A.Ü.T.F. İnsan Araştırmaları Etik Kurulu
Aslı Gibidir

CamScanner ile tarandı
