# Supplementary material for: The efficacy of suprascapular nerve radiofrequency and intra-articular steroıd injectıon in frozen shoulder treatment: randomızed controlled trial
Source: BMC Anesthesiol. 2026 Aug 13;26:663. doi: 10.1186/s12871-026-04162-7 (PMC13749883; doi:10.1186/s12871-026-04162-7)
Supplement: Supplementary file 4 [file 12871_2026_4162_MOESM3_ESM.docx]

**TİTLE PAGE**

**Manuscrpit title:** THE EFFICACY OF SUPRASCAPULAR NERVE RADIOFREQUENCY AND INTRA-ARTICULAR STEROID INJECTION IN FROZEN SHOULDER TREATMENT: RANDOMIZED CONTROLLED TRIAL

**Authors’ information**

**Name and Surname:** Dostali ALİYEV

**Title:** Ass. Prof.

**ORCID number:** [0000-0002-6111-0938](https://orcid.org/0000-0002-6111-0938)

**E-mail address:** [daliyev@tobbetuhastanesi.com.tr](mailto:daliyev@tobbetuhastanesi.com.tr)

**İnstitution:** Tobb Etü Hospital, Faculty of Medicine, Department of Anesthesiology and

Reanimation, Division of Algology, Ankara, Turkey

**Name and Surname:** İbrahim AŞIK

**Title:** Prof. Dr.

**ORCID number:** 0000-0002-4800-3150

**E-mail address:** [alidostaz@yahoo.com](mailto:alidostaz@yahoo.com)

**İnstitution:** Ankara University, Faculty of Medicine, Department of Anesthesiology and

Reanimation, Division of Algology, Ankara, Turkey

**Running title:** THE EFFICACY OF RADIOFREQUENCY IN FROZEN SHOULDER

**Corresponding author**

**Name and surname:** Dostali ALİYEV

**Address:** Beştepe, Yaşam Cd. No:5, 06560 Yenimahalle/Ankara

**Telephone:** 090 551 435 19 36

**E-mail address:** [daliyev@tobbetuhastanesi.com.tr](mailto:daliyev@tobbetuhastanesi.com.tr)

**Previous presentation in conferences:** None

**Conflict of interest:** The authors declare that they have no conflict of interest

**There is no funding support for the study**

**Data availability**: The data that support the findings of this study are available on request from the corresponding author.

**Patient consent statement:**All participants provided written informed consent after receiving detailed information about the study.

**Study Center:**Ankara University, Faculty of Medicine, Department of Anesthesiology and

Reanimation, Division of Algology, Ankara, Turkey

**IRB number:** İ7-492-21

**U.S. Clinical Trial Registry No:** NCT06778577

**Significance:** This randomized controlled trial fills an important gap in the literature by being the first to evaluate the combined use of **suprascapular nerve pulsed radiofrequency and intra-articular steroid injection** in patients with adhesive capsulitis. The findings demonstrate **earlier and significantly greater improvements in pain relief, shoulder function, and range of motion** compared with intra-articular injection alone, highlighting the clinical superiority of the combined approach. By presenting a **safe, ultrasound-guided, and minimally invasive strategy with clear functional benefits**, this study provides valuable evidence that may contribute to optimizing contemporary management of frozen shoulder.
