# Supplementary material for: The efficacy of suprascapular nerve radiofrequency and intra-articular steroıd injectıon in frozen shoulder treatment: randomızed controlled trial
Source: BMC Anesthesiol. 2026 Aug 13;26:663. doi: 10.1186/s12871-026-04162-7 (PMC13749883; doi:10.1186/s12871-026-04162-7)
Supplement: Supplementary file 5 [file 12871_2026_4162_MOESM5_ESM.docx]

COVER LETTER

Date: 12.02.2026

Dear Editor,

Please find enclosed our manuscript, “The Efficacy Of Suprascapular Nerve Radiofrequency And Intra-Articular Steroid Injection In Frozen Shoulder Treatment: Randomized Controlled Trial

” by Dostali ALİYEV et al., which we would like to submit for publication as an original article in ‘BMC Anesthesiology ’.

Thank you for your time and assistance.

Sincerely,

Corresponding author: Dostali ALİYEV

Mailing address: [dr.dostalialiyev@gmail.com](mailto:dr.dostalialiyev@gmail.com)

Contact phone number: +90 551 435 19 36
